# Anti-inflammatory effects of thymol: an emphasis on the molecular interactions through *in vivo* approach and molecular dynamic simulations

**DOI:** 10.3389/fchem.2024.1376783

**Published:** 2024-06-25

**Authors:** Muhammad Torequl Islam, Mehedi Hasan Bappi, Md Shimul Bhuia, Siddique Akber Ansari, Irfan Aamer Ansari, Manik Chanda Shill, Tala Albayouk, Na’il Saleh, Mohamed El-Shazly, Heba A. S. El-Nashar

**Affiliations:** ^1^ Department of Pharmacy, Bangabandhu Sheikh Mujibur Rahman Science and Technology University, Gopalganj, Bangladesh; ^2^ BioLuster Research Center, Dhaka, Bangladesh; ^3^ Pharmacy Discipline, Khulna University, Khulna, Bangladesh; ^4^ Department of Pharmaceutical Chemistry, College of Pharmacy, King Saud University, Riyadh, Saudi Arabia; ^5^ Department of Drug Science and Technology, University of Turin, Turin, Italy; ^6^ Department of Pharmaceutical Sciences, North South University, Dhaka, Bangladesh; ^7^ Department of Chemistry, College of Science, United Arab Emirates University, Al Ain, United Arab Emirates; ^8^ Department of Pharmacognosy, Faculty of Pharmacy, Ain Shams University, Cairo, Egypt

**Keywords:** cyclooxygenase, in silico studies, inflammation, oxidative stress, thymol

## Abstract

Thymol (THY), as the natural monoterpene phenol, acts against oxidative stress and inflammatory processes. This study aimed to evaluate the anti-inflammatory effects and possible molecular mechanisms of THY via formalin-induced mouse and egg albumin-induced chick models alongside molecular docking and molecular dynamic (MD) simulations. THY (7.5, 15, and 30 mg/kg) was investigated, compared to celecoxib and ketoprofen (42 mg/kg), as anti-inflammatory standards. THY dose-dependently and significantly (*p* < 0.05) decreased paw-licking and edema diameter parameters in formalin (phases I and II) and egg albumin-induced models. Moreover, THY (15 mg/kg) exerted better anti-inflammatory effects in combination with the standard drug ketoprofen than alone and with celecoxib. In silico studies demonstrated elevated binding affinities of THY with cyclooxygenase-2 (COX-2) than the COX-1 enzyme, and the ligand binds at a similar location where ketoprofen and celecoxib interact. The results of MD simulations confirmed the stability of the test ligand. THY exerted anti-inflammatory effects on Swiss mice and young chicks, possibly by interacting with COX-2. As a conclusion, THY might be a hopeful drug candidate for the management of inflammatory disorders.

## Graphical Abstract|

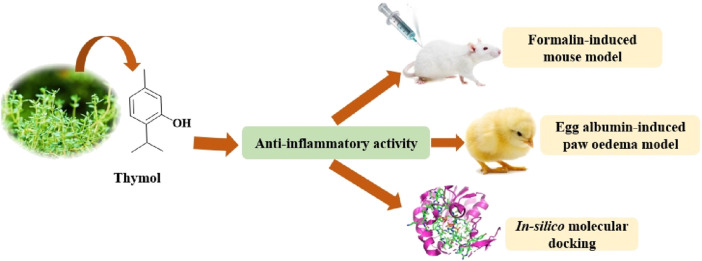

## 1 Introduction

Inflammation connects a wide variety of pathophysiological processes. Although inflammation plays many important physiological roles, it is one of the major drivers of many diseases, including cancer (Singh et al., 2019; El-Nashar et al., 2021). Pathogenic microbes such as bacteria, fungi, or viruses usually invade our bodies and result in infection and inflammation (Isailovic et al., 2015; El-Nashar et al., 2022). Non-steroidal anti-inflammatory drugs (NSAIDs) are frequently used to treat inflammation (Rabie et al., 2023b). It is due to their promising efficacy in the management of inflammation and pain (Jamaddar et al., 2023). Besides the anti-inflammatory effect, most NSAIDs have analgesic and antipyretic properties (Rabie et al., 2023a). Cumulative studies suggest that commonly used NSAIDs exert adverse effects on our digestive tract, heart, liver, kidneys, and brain (Bacchi et al., 2012; Bindu et al., 2020). Therefore, searching for safer and more efficacious anti-inflammatory drugs might be a hopeful and alternative strategy.

Natural products and their derivatives are promising sources of anti-inflammatory agents (Hou et al., 2020; Ashmawy et al., 2023; Abdelazim et al., 2024). It may be due to their promising anti-microbial properties as well as the inhibitory or neutralizing capacity of pro-inflammatory and inflammatory mediators (Azab et al., 2016). Moreover, senescent cells (SCs) are evidently accumulating in tissues, thereby resulting in a loss of tissue repair ability due to the arresting cell cycle in progenitor cells, which upregulates pro-inflammatory and inflammatory markers in humans. Many natural products, for example, curcumin and its analogs, fisetin, piperlongumine, and quercetin, have senolytic properties that can protect against this pathological phenomenon (Li et al., 2019).

Thymol (THY) chemically identified as two-isopropyl-5-methylphenol is a monoterpene phenol. It has been extracted from the essential oils of many thyme species, including Coridothymus capitatus, Thymus vulgaris, and Origanum vulgare (Hazzit et al., 2006). Cumulative studies suggest that THY possesses diverse biological effects, including anti-microbial, antioxidant, anti-inflammatory, and anti-cancer activity (Salehi et al., 2018; Islam et al., 2019). THY is considered a safe and effective food supplement due to the fact that up to 500 mg/kg oral doses of this natural phenol did not show toxicity in animals (Geyikoglu et al., 2018). Moreover, it is evident that it can improve the integrity of the digestive tract and cure intestinal injury by inducing immunomodulatory and antioxidative effects (Du et al., 2016).

This study aims to evaluate the anti-inflammatory effects of thymol in Swiss mice and chicks. Additionally, the possible anti-inflammatory mechanism of this phenol derivative has also been evaluated using a conventional co-treatment strategy as well as *in silico* studies.

## 2 Materials and methods

### 2.1 *In vivo* protocols

#### 2.1.1 Chemicals and reagents

The test drug thymol (THY) was purchased from Sigma-Aldrich (Germany), while the emulsifier tween-80 and edema inducer formaldehyde were purchased from Merck (India) Co. Ltd. Celecoxib (CXB) and ketoprofen (KPN) were kindly provided by Incepta Pharmaceuticals Ltd., Bangladesh. Another edema-inducer, egg albumin, was collected from a fresh egg purchased from the local market in Gopalganj, Bangladesh.

#### 2.1.2 Selection and preparation of test/control groups

For mice, the highest test dose of THY was selected as 30 mg/kg (Liu et al., 2022), which was then serially diluted to middle and lower doses of 15 and 7.5 mg/kg, respectively. The same doses of THY were also administered to chicks for this study. Doses of the reference drugs were determined according to the method described by Shin et al. (2010) as the following equation. Finally, the middle dose of THY was co-treated with the reference drugs used in this study to see the possible modulatory effects of thymol with these drugs (Shin et al., 2010). Animal dose (mg/kg) = HED (mg/kg) × Conversion factor.where, the conversion factor for mice is 12.33.

#### 2.1.3 Experimental animals

For this study, chicks (Gallus *gallus domesticus*) of either sex having a body weight (b.w.) range of 40–42 g at 2-days-old were purchased from Nourish Grand Parent Ltd. in Rangpur, Bangladesh, while adult Swiss albino mice (24–30 g, b. w.) of either sex were purchased from the animal house of Jahangirnagar University, Savar, Bangladesh. These animals were maintained at the Pharmacology Lab of Bangabandhu Sheikh Mujibur Rahman Science and Technology University (BSMRSTU), Gopalganj. The animals were allowed free access to standard food and water *ad libitum*. They were kept under controlled lighting (12 h dark/light cycle) at 27 ± 1°C until the test commenced. The present experiment was conducted from 08:00 a.m. to 3:00 p.m., and the animals were monitored for an additional 17 h to check their possible mortality after the study. Experimental design and procedures were approved by the Department of Pharmacy at the BSMRSTU (#bsmrstu/phr1-1136/23).

#### 2.1.4 Study design

##### 2.1.4.1 Formalin-induced paw edema in mice

This study was done according to the method described by Tjølsen et al. (1992), with modifications (Tjølsen et al., 1992). Briefly, we used 12-h fasted animals after 7-day acclimation in the laboratory environment. Briefly, a total of forty mice (24–30 g, b. w.) of either sex were randomly distributed into different groups, each containing five animals (Table 1). All the treatments were given via oral gavage (p.o.) 30 minutes prior to the injection of a formalin (0.5% formaldehyde) solution in the sub-plantar area of the right hind paw of the animals. For this, 50 μL of a formalin solution prepared in normal saline was injected into each animal. Then the licking behavior was counted for the first 0–10 min (early phase: Phase I) and 20–30 min (late phase: Phase II), and the paw edema diameter was measured by using a slide caliper at 60, 90, and 120 min after formalin injection. Paw edema was determined in comparison to the baseline (normal) paw diameter of each animal in millimeters. Then the following parameters were determined:

**TABLE 1:** The treatment groups, doses, and the target receptor.

Treatment group	Composition	Dose	Target receptor
Gr-I	Vehicle (0.5% Tween 80 dissolved in normal saline)	10 mL/kg	-
Gr-II	Thymol (THY)	7.5 mg/kg	Under investigation
Gr-III		15 mg/kg	
Gr-IV		30 mg/kg	
Gr-V	Celecoxib (CXB)	42 mg/kg	COX-2
Gr-VI	Ketoprofen (KPN)	42 mg/kg	COX-1, COX-2
Gr-VII	THY-15 + CXB-42	15 mg/kg + 42 mg/kg	Under investigation
Gr-VIII	THY-15 + KPN-42	15 mg/kg + 42 mg/kg	Under investigation

All treatments are given at 10 mL/kg via oral gavage (p.o.).

PED (mm) = Paw diameter in observed time – Baseline paw diameter

$$\%\text{RPE}=\left[\left({\text{PDOT}}_{\text{Vehicle}}\;–\;{\text{PDOT}}_{\text{Test}}\right)÷{\text{PDOT}}_{\text{Vehicle}}\right]\times 100$$

%TRE: (Number of animals with normal paw ÷ Total animals in the group) × 100.where, PED, PDOT, RPE, and TRE mean paw edema diameter, paw diameter in observed time, reduction of paw edema, and total reduction of edema at final observation time.

##### 2.1.4.2 Egg albumin-induced paw edema in chicks

A total of thirty chicks (45–50 g, b. w.; 4 days old of either sex) have fasted for 2 h (except water). Then the chicks were randomly divided into the following groups:

Gr-I: Vehicle (0.5% tween-80 dissolved in normal saline); Gr-II: THY 15 mg/kg; Gr-III: CXB 42 mg/kg; Gr-IV: KPN 42 mg/kg; Gr-V: THY-15 + CXB-42: THY 15 mg/kg + CXB 42 mg/kg; Gr-VI: THY-15 + KPN-42: THY 15 mg/kg + KPN 42 mg/kg.

Thirty minutes after the above-mentioned treatments, 100 µL of egg albumin (1% w/v in saline solution) was injected into the sub-plantar tissue of the right hind paw of each chick. Then the PED (mm), %RPE, and %TRE were determined.

#### 2.1.5 Statistical analysis

The results are presented as Mean ± S.E.M. (standard error of the mean) or percentage. The data were analyzed by means of the analysis of variance (ANOVA) followed by the t-Student–Newman-Keuls post-hoc test using the statistical software GraphPad Prism (version 6.5), and the experimental groups were compared with the vehicle (control) group. The levels of statistical significance ranged from *p* < 0.05 at 95% confidence intervals.

### 2.2 In silico study

#### 2.2.1 Preparation of proteins and active site prediction

We have selected two proteins (COX-1 and COX-2) based on published literature that are associated with formalin-induced inflammatory reactions to show the ligand-receptor binding interaction by performing molecular docking studies (Ahmad et al., 2020; Huang et al., 2022). The following proteins’ 3D structures were made available by the RCSB Protein Data Bank: COX-1 (6Y3C) is bound with 2-hydroxypropane-1,2,3-tricarboxylate as a co-crystal ligand, and COX-2 (3LN1) is found with celecoxib as a co-crystal ligand (https://www.rcsb.org, accessed on 27 January 2024). The CASTp web tool was utilized for the identification of active sites in 14 enzymes. This was achieved through the implementation of the pocket algorithm derived from the alpha shape theory as well as incorporating the latest theoretical findings from the field of computational geometry (Chowdhury et al., 2024b). The PyMol software program (v2.5.8) was then used to carefully optimize the collected protein sequence in order to eliminate any extraneous macromolecules and molecules, including proteins, co-crystal ligands, lipids, heteroatoms, and water molecules (Bhuia et al., 2023a). Lastly, energy was minimized, and protein structure was optimized using the SwissPDB Viewer computer program. This phase included using the GROMOS96 force field, and the resultant PDB file was stored for further molecular docking studies (Bhuia et al., 2023b).

#### 2.2.2 Collection and preparation of ligands

**FIGURE 1:**
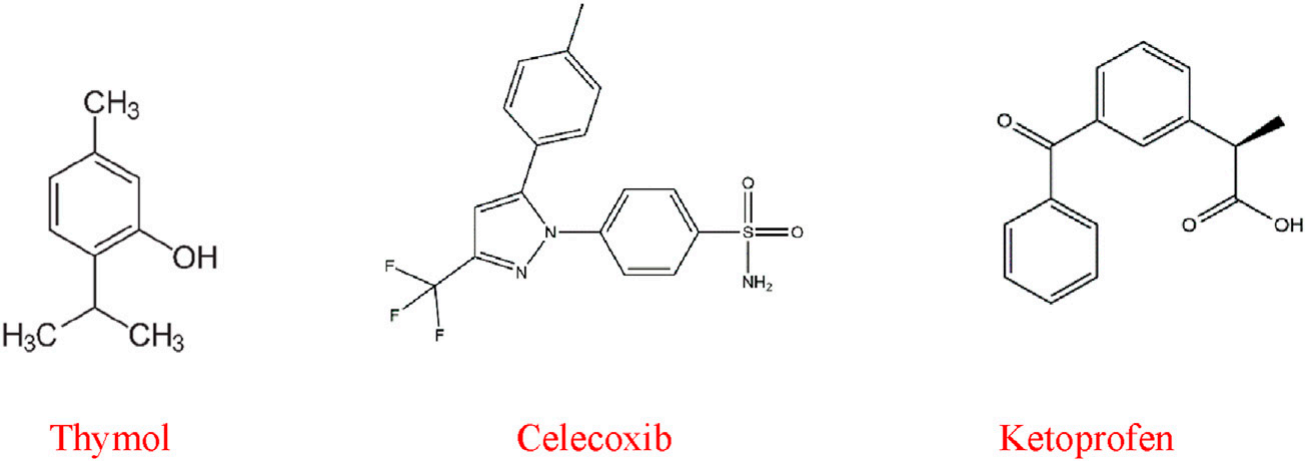
2D structure of thymol and reference drugs.

We selected two well-known and readily available anti-inflammatory drugs as reference ligands based on the literature. Our goal was to compare the molecular interaction and binding affinity of these drugs with the selected test ligand (AA). The 3D structures of the test and reference ligands of thymol (Compound CID: 6989), celecoxib (compound CID: 2662), and ketoprofen (compound CID: 3825) are gathered in SDF format from the chemical database PubChem (https://pubchem.ncbi.nlm.nih.gov/). The energy minimization of the selected ligands was done using the Chem3D 16.0 computer program. Next, the minimized ligands were stored as SDF files so that they could be prepared for the molecular docking process (Chowdhury et al., 2024a). The 2D chemical structures of the ligands are shown in Figure 1.

#### 2.2.3 Molecular docking study

To estimate the required energy of a ligand for interacting with the active sites toward its receptor, molecular docking was performed, which was done using the PyRx software tool. In order to ensure effective docking, the grid box dimensions were set at 34.93 × 28.97 × 9.57 Å along the x-, y-, and *z*-axes, respectively. The docking calculation was done in two thousand steps (Chowdhury et al., 2023). The ligand-protein complex is assembled in PDB format. The docking possibilities result (docking affinity) was saved in ‘csv’ format. The interactions that take place between ligand-proteins and the active site of the receptor were visualized using PyMol (v2.5.8) and Discovery Studio Visualizer (v21.1.020298). The types of bonds, total number and length of hydrogen bonds (HBs), and amino acid residues associated with every interaction between a ligand and a protein are then documented.

#### 2.2.4 Molecular dynamic simulations

To determine the stability of the targeted protein (COX-2) interaction with the selected ligand molecules (CXB and THY) was chosen and subjected to 50 nanoseconds MD simulations. The “Desmond v3.6 Program” from Schrodinger (https://www.schrodinger.com/ac) (Academic version) was used to model the molecular dynamics of the protein-ligand complex structures (Omar et al., 2022). In order to create the intended framework, a pre-determined TIP3P water method was developed to construct a precise volume with periodic orthorhombic coordinates spaced 10 mm apart. The requisite ions, such as 0+ and 0.15 M salts, were randomly introduced into the solvent solution in order to achieve electrical neutrality within the framework. The solvency protein system was developed by employing a ligand complex, and the system construction was simplified using the default protocol. The application of OPLS3e force field parameters within the Desmond module enabled the complete accomplishment of this task (Ahammad et al., 2021). The NPT assemblies were conducted under standard conditions of 101,325 bar (1 atm) pressure and 300 K temperature. Prior to the assemblies, there were 50 PS capture sessions that resulted in a total energy of 1.2 kcal/mol. The Nose-Hoover temperature coupling method and the isotropic approach were employed in these assemblies. The screenshots of the MD simulation were generated using Schrodinger’s maestro application, version 9.5. The Simulations interaction diagram, developed from the Desmond modules of the Schrodinger suite, has been utilized to examine the simulation event and evaluate the reliability of the MD simulation. The stability of the protein-ligand complex structures was assessed by analyzing various factors including trajectory performance, root mean square fluctuation (RMSF), root mean square deviation (RMSD), solvent accessible surface area (SASA), intramolecular hydrogen bonds, radius of gyration (Rg), protein-ligand contacts (PL), polar surface area (PSA), and MolSA. The root mean square deviation (RMSD) in molecular dynamics simulations is the average distance traveled by an atom over a specific time period compared to a reference time (Abdullah et al., 2023). The RMSD of the structural atoms of a protein, including heavy particles and backbone, was initially determined. This was followed by measuring the RMSD of protein-bound ligand compounds at various time intervals, which were realigned and compared to the reference time (in our study, 50 ns). The following equation (Eq. (1)) can be used to determine the RMSD of an MD simulation concerning the period of x:

$$\text{RMSD}=\sqrt{\frac{\sum_{i=0}^{N}\left[m_{i}\times {\left(X_{i}-Y_{i}\right)}^{2}\right]}{M}}$$ (1)

N represents the quantity of chosen atoms, rʹ denotes the position of the bit in system x after the reference system’s point has been aligned, and j signifies the reference time. The root mean square fluctuation (RMSF) is a regularly used method to detect and track local changes in the conformational shape of proteins (Martínez, 2015). The RMSF estimation of a MD’s simulation of a protein with two residues can be obtained using the continuity equation.

$$\text{RMSF}=\sqrt{\frac{1}{T}\sum_{T_{j}}^{T}{\left(X_{i}\left(t_{j}\right)-x_{j}\right)}^{2}}$$ (2)

## 3 Results

### 3.1 *In vivo* studies

#### 3.1.1 Formalin-induced paw edema in mice

According to Table 2, in the early phase (Phase I), both test and/or standard groups in comparison to the vehicle group (Gr-I) significantly (*p* < 0.05) reduced the number of licks in animals. THY dose-dependently reduced the licking parameters. THY at all doses produced better effects than the standard drug CXB (18.20 ± 2.30). Besides this, THY at 15 (7.20 ± 0.74) and 30 mg/kg (6.40 ± 0.84) also showed better effects than the KPN (7.80 ± 2.92). Between the standard drugs, KPN (Gr-VI) reduced the number of licks better than CXB (Gr-V). THY 15 mg/kg (Gr-III) co-administered with KPN 42 mg/kg (Gr-VI) also produced better results than CXB 42 mg/kg (Gr-VII). THY and/or standard drugs also significantly reduced the number of paw-lickings in the late phase (Phase II). THY-30 (Gr-IV) and THY-15 + KPN-42 (Gr-VIII) reduced licking parameters prominently in comparison to the other groups. In this case, THY at 15 and 30 mg/kg also exerted better effects than the standard drugs. KPN-42 alone (Gr-VI) or its combination (Gr-VIII) produced better effects than CXB-42 alone (Gr-V) or its combination (Gr-VII). In comparison to Phase I, a significant reduction in paw-licking behavior was observed in Phase II.

**TABLE 2:** Number of paw-licking observed in test and/or control groups of formalin induced inflammatory test.

Treatment groups	Paw-licking	
	Phase I (*early phase*)	Phase II (*late phase*)
Gr-I	44.40 ± 2.51	18.20 ± 2.10
Gr-II	9.80 ± 0.42^*b^	3.60 ± 0.57^*b^
Gr-III	7.20 ± 0.74^*bc^	2.20 ± 0.42^*b^
Gr-IV	6.40 ± 0.84^*abc^	1.60 ± 0.57^*abc^
Gr-V	18.20 ± 2.30^*^	9.40 ± 0.57^*^
Gr-VI	7.80 ± 2.92^*b^	2.40 ± 0.57^*b^
Gr-VII	6.60 ± 1.04^*abc^	5.20 ± 0.42^*b^
Gr-VIII	5.60 ± 0.91^*abc^	1.20 ± 0.65^*abc^

Values are Mean ± SEM (standard error of the mean) (n = 5); One-way ANOVA, followed by *t*-Student–Newman–Keuls’s as post-hoc test; ^*^ *p* < 0.05 compared to the Vehicle group; ^a^ *p* < 0.05 compared to the Gr-III; ^b^ *p* <0.05 compared to the Gr-V; ^c^ *p* <0.05 compared to the Gr-VI; Gr-I: vehicle; Gr-II: Thymol (THY) 7.5 mg/kg; Gr-III: THY, 75 mg/kg; Gr-IV: THY, 30 mg/kg; Gr-V: Celecoxib (CXB) 42 mg/kg; Gr-VI: Ketoprofen (KPN) 42 mg/kg; Gr-VII: THY-15 + CXB-42; Gr-VIII: THY-15 + KPN-42.

Table 3 suggests that all the treatment groups (alone or in combination) significantly reduced edema diameter at all observation times. In all cases, THY-15 + KPN-42 reduced edema diameter significantly (*p* < 0.05) compared to their alone and other treatment groups. At 2 h of observation time, THY all doses and its combination groups exerted better than the first two observation periods. THY also showed a dose-dependent %TRE, where its highest dose (30 mg/kg) exhibited a %TRE (80.00%) similar to that of Gr-VIII (THY-15 + KPN-42). Gr-V (CXB-42) did not show %TRE; however, when co-treated with THY-15, it showed %TRE by 60%, suggesting a THY-mediated synergistic effect in edematous animals.

**TABLE 3:** Paw edema diameter observed in test and/or control groups of formalin induced inflammatory test.

Treatment group	Edema diameter (mm)			TRE (%)
	60 min	90 min	120 min	
Gr-I	2.20 ± 0.22	1.80 ± 0.22	1.80 ± 0.22	0.00
Gr-II	1.80 ± 0.14^*^	1.10 ± 0.11^*b^	0.70 ± 0.14^*b^	0.00
Gr-III	1.80 ± 0.38^*^	1.00 ± 0.18^*b^	0.50 ± 0.25^*bc^	40.00^*b^
Gr-IV	1.70 ± 0.22^*a^	0.90 ± 0.12^*abc^	0.20 ± 0.22^*abc^	80.00^*bc^
Gr-V	1.60 ± 0.27^*a^	1.40 ± 0.27^*^	1.00 ± 0.00^*^	0.00
Gr-VI	1.60 ± 0.27^*a^	1.00 ± 0.00^*b^	0.60 ± 0.27^*b^	40.00^*b^
Gr-VII	1.90 ± 0.37^*^	1.10 ± 0.27^*b^	0.40 ± 0.27^*abc^	60.00^*bc^
Gr-VIII	1.50 ± 0.35^*abc^	0.70 ± 0.14^*abc^	0.20 ± 0.22^*abc^	80.00^*bc^

Values are Mean ± SEM (standard error of the mean) (n = 5); One-way ANOVA, followed by t-Student–Newman–Keuls’s as post-hoc test; *p < 0.05 compared to the Vehicle group; ^a^ *p* < 0.05 compared to the Gr-III; ^b^ *p* <0.05 compared to the Gr-V; ^c^ *p* <0.05 compared to the Gr-VI; Gr-I: vehicle; Gr-II: Thymol (THY) 7.5 mg/kg; Gr-III: THY, 75 mg/kg; Gr-IV: THY, 30 mg/kg; Gr-V: Celecoxib (CXB) 42 mg/kg; Gr-VI: Ketoprofen (KPN) 42 mg/kg; Gr-VII: THY-15 + CXB-42; Gr-VIII: THY-15 + KPN-42.

**FIGURE 2:**
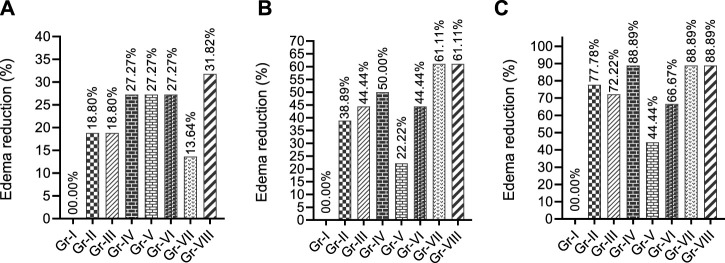
Paw edema reduction profiles of test and/or control groups in formalin induced model observed different observatory periods **(A)** 60 min, **(B)** 90 min and **(C)** 120 min [Values are expressed as percentages in comparison to the vehicle group; TRE: Total reduction of edema at final observation time. Gr-I: Vehicle; Gr-II: Thymol (THY) 7.5 mg/kg; Gr-III: THY 75 mg/kg; Gr-IV: THY 30 mg/kg; Gr-V: Celecoxib (CXB) 42 mg/kg; Gr-VI: Ketoprofen (KPN) 42 mg/kg; Gr-VII: THY-15 + CXB-42; Gr-VIII: THY-15 + KPN-42.

The percentage RPE shown in Fig. 2 suggests that THY at 7.5 and 15 mg/kg (Gr-II and III) reduced paw edema by 18.18%, while its 30 mg/kg dose reduced it by 27.27% after 1 h in comparison to the Gr-I. Both standard drugs (Gr-V and VI) exhibited similar %RPE. THY-15 co-treated with KPN (Gr-VIII) showed a better %RPE than all the other groups at the first observation time (60 min). In the second observation period (90 min), THY dose-dependently reduced paw edema in mice. At all doses, it showed a better %RPE than the CXB (Gr-V), while at 15 mg/kg, it produced a similar effect that the KPN (Gr-VI) showed. However, co-treatment groups (Gr-VII and VIII) showed the highest %RPE values (61.11%) of all treated groups. Inspecting the third observation time (2 h), THY also significantly reduced edema volume in animals, where at 30 mg/kg it showed %RPE similar to the co-treatment groups (88.89%). This figure (Figure 2) indicates that all the treatment groups increased %RPE in a time-dependent manner.

#### 3.1.2 Egg albumin-induced paw edema in chicks

Table 4 suggests that test and/or standard drugs significantly (*p* < 0.05) reduced the number of licks in animals in comparison to the control group (Gr-I). THY-15 (Gr-II) exerted a better effect than the standard drug CXB (Gr-III); it exerted an effect like KPN (Gr-IV). THY-15 co-treated with CXB-42 or KPN-42 resulted in better effects than the individual groups of these reference drugs. However, THY-15 exerted better-combined effects with KPN (3.58 ± 1.54) than the CXB (6.20 ± 1.91).

**TABLE 4:** Number of paw-licking observed in test and/or control groups in egg albumin-induced chick model.

Treatment group	Licking
Gr-I	24.40 ± 3.31
Gr-II	6.60 ± 2.78^*b^
Gr-III	9.20 ± 2.51^*^
Gr-IV	5.40 ± 1.90^*ab^
Gr-V	6.20 ± 1.91^*ab^
Gr-VI	3.58 ± 1.54^*abc^

Values are Mean ± SEM (standard error of the mean) (n = 5); One-way ANOVA, followed by *t*-Student–Newman–Keuls’s as post-hoc test; ^*^ *p* < 0.05 compared to the Vehicle group; ^a^ *p* < 0.05 compared to the Gr-II; ^b^ *p* <0.05 compared to the Gr-III; ^c^ *p* <0.05 compared to the Gr-IV; Gr-I: vehicle; Gr-II: THY, 15 mg/kg; Gr-III: Celecoxib (CXB) 42 mg/kg; Gr-IV: Ketoprofen (KPN) 42 mg/kg; Gr-V: THY-15 + CXB-42; Gr-VI: THY-15 + KPN-42.

Table 5 suggests that all the treatment groups (alone or in their combinations) significantly reduced edema diameter at all observation times. In all cases, THY-15 + KPN-42 reduced edema diameter significantly (*p* < 0.05) compared to their alone and other treatment groups. At 1.5 h of observation time, THY all doses and its combination groups exerted better than the first (1 h) and third (2 h) observation periods. THY at 15 mg/kg showed 40.00% TRE, while Gr-III (CXB-42) and Gr-IV (KPN-42) showed 20.00% and 40.00%, respectively. However, THY-15, when combined with these reference drugs, CXB-42 and KPN-42, significantly increased the %TRE by 50% and 100%, respectively, suggesting a THY-mediated synergistic effect in edematous animals.

**TABLE 5:** Paw edema diameter observed in test and/or control groups in egg albumin-induced chick model.

Treatment group	Edema diameter (mm)			TRE (%)
	60 min	90 min	120 min	120 min
Gr-I	2.15 ± 0.15	2.20 ± 0.15	2.25 ± 0.25	0.00
Gr-II	1.65 ± 0.35^*b^	1.20 ± 0.15^*b^	0.55 ± 0.15^*bc^	40.00^*b^
Gr-III	1.75 ± 0.25^*^	1.60 ± 0.17^*^	1.10 ± 0.09^*^	20.00^*^
Gr-IV	1.55 ± 0.21^*ab^	1.10 ± 0.09^*ab^	0.65 ± 0.17^*b^	40.00^*b^
Gr-V	1.70 ± 0.31^*^	1.45 ± 0.25^*b^	0.85 ± 0.12^*b^	60.00^*abc^
Gr-VI	1.35 ± 0.26^*abc^	0.75 ± 0.19^*abc^	0.15 ± 0.08^*abc^	80.00^*abc^

Values are Mean ± SEM (standard error of the mean) (n = 5); One-way ANOVA, followed by *t*-Student–Newman–Keuls’s as post-hoc test; ^*^ *p* < 0.05 compared to the Vehicle group; ^a^ *p* < 0.05 compared to the Gr-II; ^b^ *p* <0.05 compared to the Gr-III; ^c^ *p* <0.05 compared to the Gr-IV; Gr-I: vehicle; Gr-II: THY, 15 mg/kg; Gr-III: Celecoxib (CXB) 42 mg/kg; Gr-IV: Ketoprofen (KPN) 42 mg/kg; Gr-V: THY-15 + CXB-42; Gr-VI: THY-15 + KPN-42.

**FIGURE 3:**
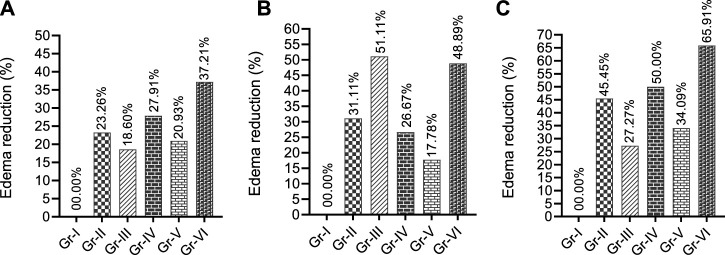
Paw edema reduction profiles of test and/or control groups in egg albumin-induced model in chicks observed different observatory periods **(A)** 60 min, **(B)** 90 min and **(C)** 120 min [Values are expressed as percentages in comparison to the vehicle group; TRE: Total reduction of edema at final observation time. Gr-I: Vehicle; Gr-II: THY 15 mg/kg; Gr-III: Celecoxib (CXB) 42 mg/kg; Gr-IV: Ketoprofen (KPN) 42 mg/kg; Gr-V: THY-15 + CXB-42; Gr-VI: THY-15 + KPN-42].

Figure 3 suggests that at the first two observation times (1 h and 1.5 h), THY or KPN alone (Gr-II and IV) and their combination (Gr-VI) effectively reduced the edema in chicks. THY exhibited a better %RPE than the CXB alone (Gr-III) or its combination (Gr-V). However, an inspection of the third observation time (2 h) of CXB alone (Gr-III) showed a better %RPE (51.11%) than its combination and other treatment groups. THY alone (Gr-II) exerted better effects (31.11%) than Gr-IV (26.67%) and Gr-V (17.78%). However, the co-treatment group THY-15 + KPN-42 (Gr-VI) significantly increased %RPE compared to their individual groups (Gr-II and IV). This figure (Figure 3) indicates that all the treatment groups increased % RPE at 1.5 h.

### 3.2 In silico studies

#### 3.2.1 Molecular docking scores of thymol and referral ligands with cyclooxygenases

We selected the CASTp web server for evaluating the enzyme active site among other predicted programs due to its distinct advantage in thoroughly examining protein surface characteristics beyond solely predicting the active site. The emphasis on characterizing cavities, pockets, and surface topology allows for a comprehensive comprehension of the protein’s structure and putative functional areas. The present investigation utilized the CASTp web server to forecast active sites for two chosen enzymes based on their dissimilar volume scores. The site with the greatest volume was chosen as the definitive active site for further study. Among the several chains of these enzymes, we saw that each chain possessed its own distinct active site. According to our *in silico* investigation, the experimental ligand (THY) interacted with the COX-1 enzyme with a docking score of −5.9 kcal/mol. In contrast, the standard ligand (KPN) revealed an elevated binding affinity of −7.9 kcal/mol toward COX-1. On the other hand, the other referral ligand CXB manifested better binding interaction with the highest docking scores of −12.2 kcal/mol, while the THY revealed a docking score of −6.7 kcal/mol toward COX-2 macromolecules. However, the comparison of THY’s binding interaction with COXs showed that THY expressed an elevated binding interaction with COX-2 with a higher docking value. The docking scores of all ligands with COX-1 and COX-2 enzymes are given in Table 6.

**TABLE 6:** Molecular docking scores of thymol and celecoxib with cyclooxygenases.

Ligands	Enzyme (PDB ID)	Docking score (kcal/mol)
THY	COX-1 (6Y3C)	−5.9
KPN		−7.9
THY	COX-2 (3LN1)	−6.7
CXB		−12.2

COX: Cyclooxygenase; THY: Thymol; KPN: Ketoprofen; CXB: Celecoxib

#### 3.2.2 Visualization of ligands-proteins interactions and binding sites

**FIGURE 4:**
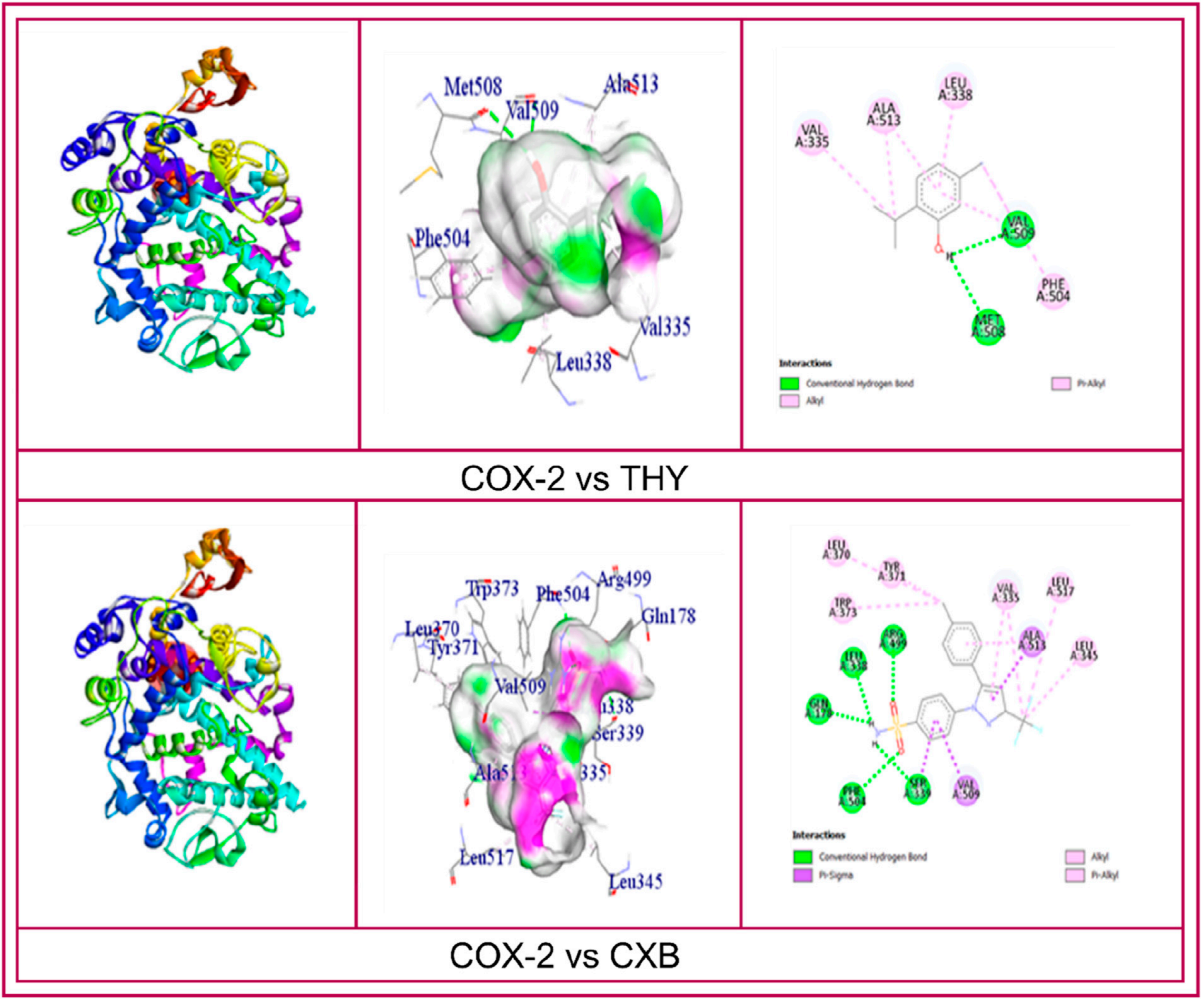
2D and 3D visualization binding sites and interacted amino acid residues of COXs enzymes with the celecoxib, ketoprofen and thymol.

CASTp prediction for the active site demonstrated that TYR371 amino acid (AA) residue is present in the active site for COX enzyme binding. However, the molecular docking investigation exhibited different types of hydrogen bonds (HBs), such as carbon, conventional HB, and various hydrophobic (HP) bonds, such as sigma, alkyl, pi-alkyl, pi-sulfur, pi-pi T-shaped, pi-cation bonds, and pi-pi stacked. The reference ligand KPN binds with the COX-1 enzyme by forming HP bonds with amino acid (AA) residues of TYR39, CYS36, CYS47, PRO153, LEU152, ARG469, and CYS41. Whereas, the tested ligand (THY) binds with COX-1 in the same binding site by forming some HP bonds with similar AA residues, including CYS41, LEU152, CYS47, and TYR39. In the case of the COX-2 enzyme, the referral ligand CXB formed five HB with AA residues of SER339, GLN178, LEU338, ARG499, and PHE504 (bond length ranging from 2.09 to 2.66) and several HP bonds with the AA of SER339, VAL509, ALA513, LEU370, VAL335, LEU345, LEU517, TYR371, and TRP373. On the other hand, the test ligand THY also binds in the same location as the CXB binding site. THY produced two HB and five HP bonds with COX-2 with the similar AA residues of the CXB binding site (Table 7). The 2D and 3D visualization binding sites of COX enzymes with the selected ligands are represented in Figure 4.

**TABLE 7:** Interacted amino acid residues, bond type and hydrogen bond distance of COXs enzymes and selected ligands.

Ligands	Protein	HB residues	HB distance (Å)	HB angle (Degree)	Other binding residues (hydrophobic)
THY	COX-1	-	-	-	CYS41 (Pi-Sulfur), LEU152 (Alkyl), CYS47 (Alkyl), LEU152 (Pi-Alkyl), TYR39 (Pi-Alkyl)
KPN		-	-	-	TYR39 (Pi-Pi T-shaped), CYS36 (Alkyl), CYS47 (Alkyl), PRO153 (Alkyl), LEU152 (Pi-Alkyl), ARG469 (Pi-Alkyl), CYS41 (Pi-Alkyl)
THY	COX-2	MET508	2.89	137.556	VAL335 (Alkyl), ALA513 (Alkyl), VAL509 (Alkyl), LEU338 (Pi-Alkyl), PHE504 (Pi-Alkyl)
		VAL509	2.47	137.311	
CXB		SER339	2.09	159.154	SER339 (Pi-Sigma), VAL509 (Pi-Sigma), ALA513 (Pi-Sigma), LEU370 (Alkyl), VAL335 (Alkyl), LEU345 (Alkyl), LEU517 (Alkyl), VAL509 (Pi-Alkyl), ALA513 (Pi-Alkyl), TYR371 (Pi-Alkyl), TRP373 (Pi-Alkyl)
		GLN178	2.34	142.168	
		LEU338	2.56	131.211	
		ARG499	2.25	152.236	
		PHE504	2.66	147.813	

THY: thymol; CXB: celecoxib; COX: cyclooxygenase; HB: hydrogen bond.

#### 3.2.3 Molecular dynamic simulation

##### 3.2.3.1 Analysis of RMSD and RMSF

**FIGURE 5:**
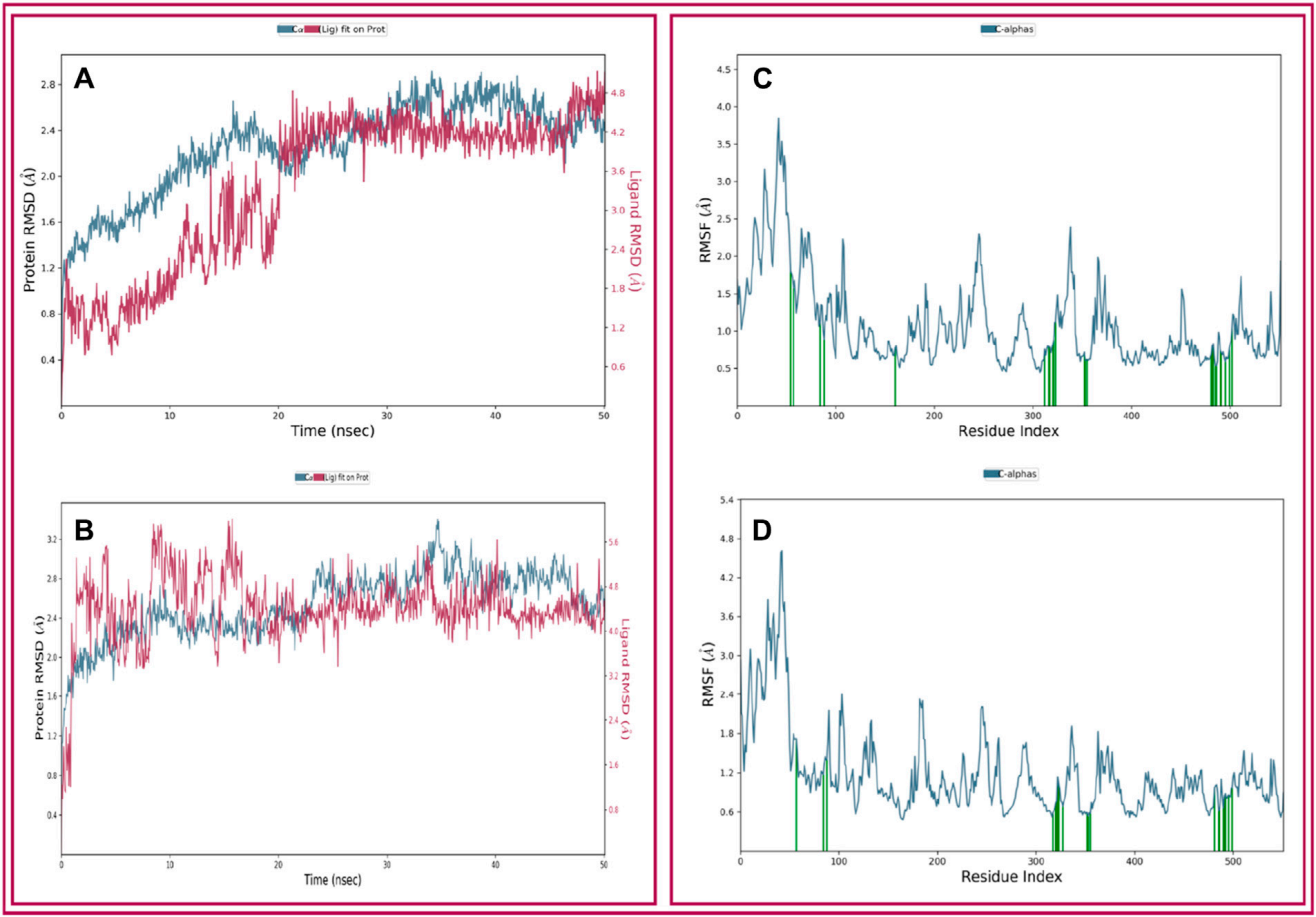
Depicted the RMSD and RMSF values extracted from the Cα atoms of the selected three compounds in complex with the COX-2 protein. Herein, showing the RMSD of COX-2 protein in complex with the compounds **(A)** celecoxib **(B)** thymol and RMSF of COX-2 protein in complex with the compounds **(C)** celecoxib and **(D)** thymol with respect to 50 ns simulation time.

The analysis of our two protein-ligand docking complexes found Cα atoms of COX-2 showed acceptable fluctuations. The values of RMSD of the selected compound (THY and CXB) complex structure have been compared with the native COX-2 protein structure to observe the changes in the order shown in Figure 5. The RMSD for the CXB-COX-2 (Figure 5A) was in a range between 0.8 and 2.8 Å, which was perfectly acceptable compared to the structure of the native protein. On the other hand, the RMSD for the THY-COX-2 complex ranges from 1.2 to 3.4 Å (Figure 5B), but the values gradually reduced and became stable at 2.4 Å after 35 ns of simulation time. In Figure 5, the ‘Lig Fit Prot’ metric calculates the RMSD of a ligand. This is done by aligning the protein-ligand complex based on the protein backbone of a reference structure and then measuring the RMSD of the ligand’s heavy atoms. The values observed are notably higher than the RMSD of the protein. In the case of CXB, the RSMD values range from 0.5 to 4.8 Å. The initial RSMD was lower, but it became higher and more stable with protein RMSD after 20 ns of simulation time. In the case of THY, the RMSD became stable after 15 ns, and no notable fluctuation was observed (Figure 5B).

The local structural fluctuations of COX-2 protein in complex with natural compound were calculated by using the deviations contributed by residues index Cα. Interestingly, residues for protein-ligands complexes have found a minimum RMSF values (Figure 5). The highest fluctuation was found for both the ligands before the 80 AA. So, analysis of RMSF and RMSD value for all protein–ligand complex supported the combined screened potential compounds.

##### 3.2.3.2 Analysis of ligands-protein contact

**FIGURE 6:**
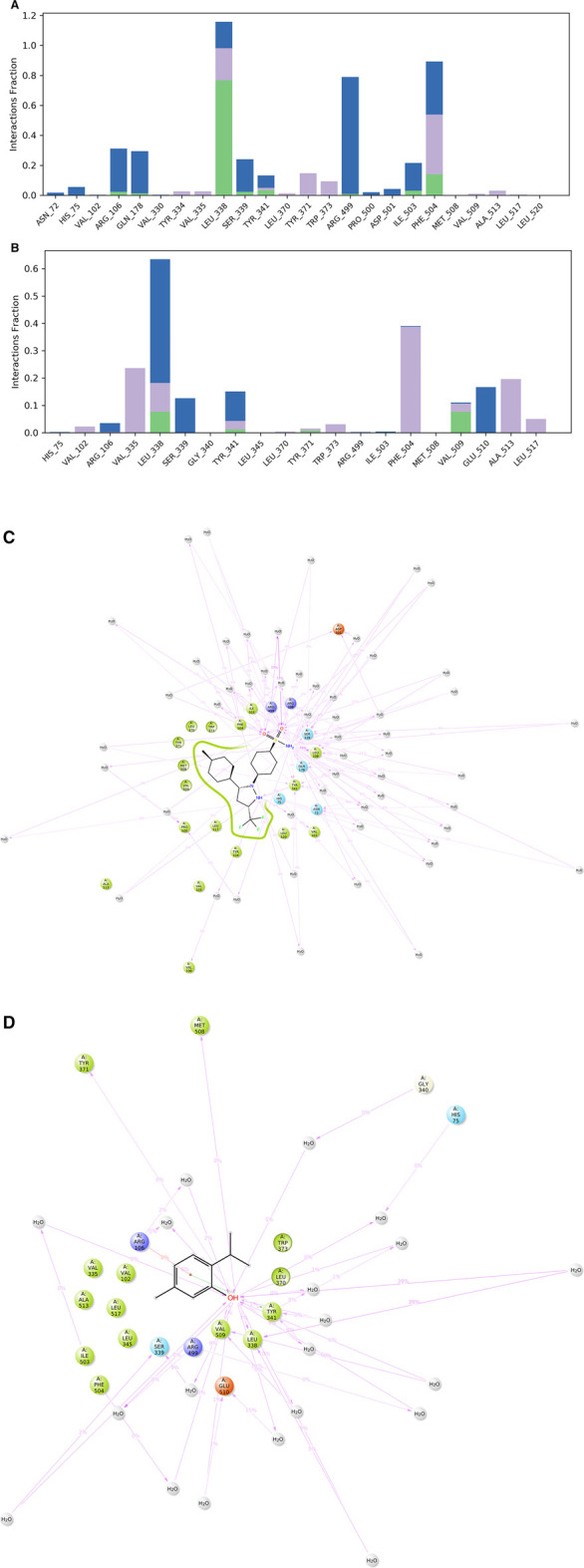
Protein–ligand contact mapping for COX-2 with the selected ligands celecoxib **(A, C)** and thymol **(B, D)** extracted from 50 ns MD simulations.

The various types of bonding interactions have a substantial impact on the binding of ligands to the intended protein. In drug design, the hydrogen-bonding properties play a crucial role in influencing the specificity, metabolization, and adsorption of drugs. The hydrogen bonds, hydrophobic, and water bridge interactions found during the MD simulation have been observed and shown in the stacked bar charts (Figure 6). Hydrogen bonding was found for all the selected ligands. CXB formed 8 HB with AA residues of ARG 106 (0.023), GLN178 (0.013), LEU338 (0.767), SER339 (0.012), TYR341 (0.032), ILE503 (0.030), and PHE504 (0.140), and the ligand also formed several hydrophobic bonds and water bridge interactions (Figure 6). On the other hand, the test ligand (THY) interacts with the selected protein through 6 HB with AA residues of ARG106 (0.001), LEU338 (0.077), TYR341 (0.012), TYR371 (0.007), MET504 (0.001), and VAL509 (0.077), as well as several hydrophobic and water bridge interactions (Figure 6).

##### 3.2.3.3 Ligand properties analysis

**FIGURE 7:**
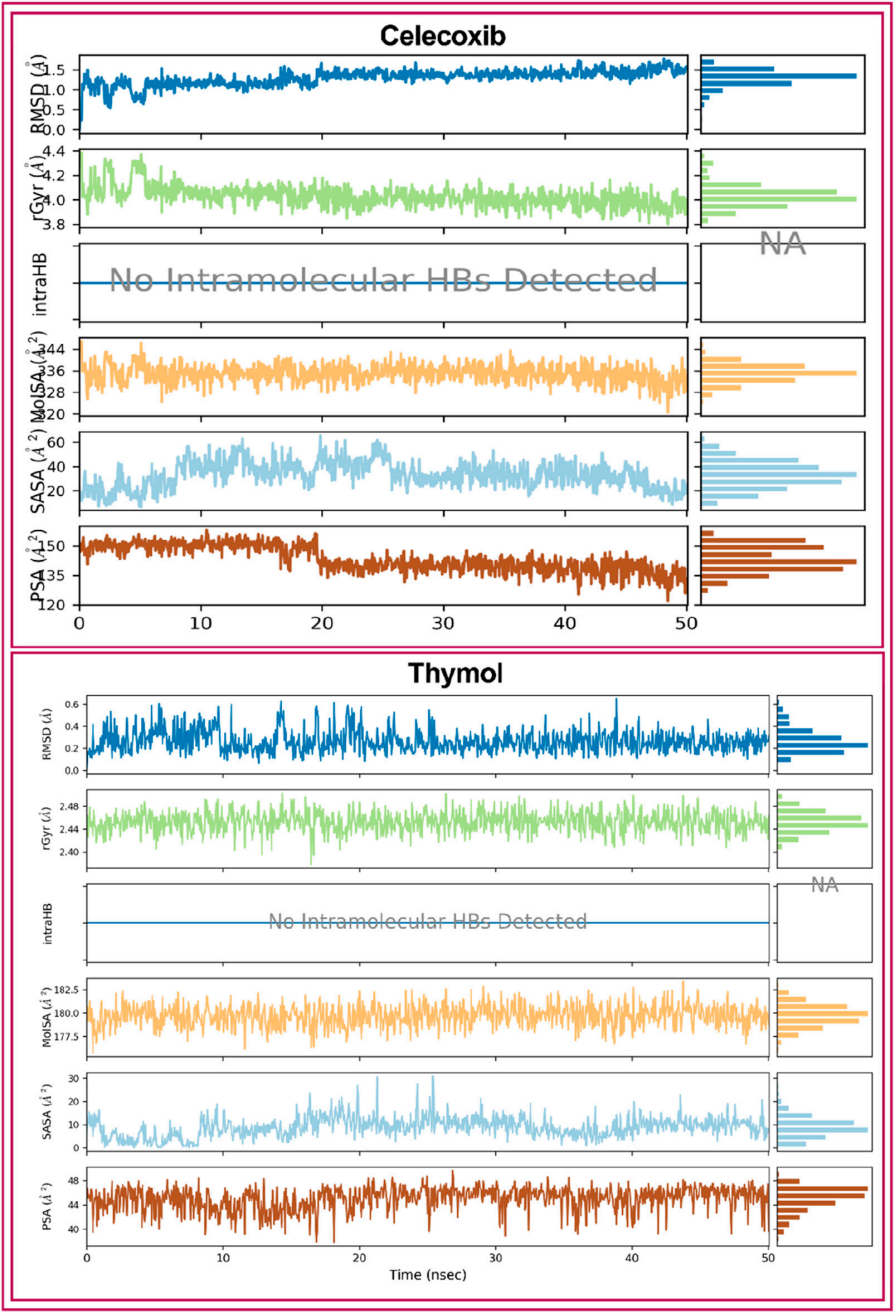
Ligands properties of the selected ligands (Celecoxib and thymol) i.e., RMSD of the ligands, Radius of Gyration (rGyr), Intramolecular Hydrogen Bonds (intraHB), Molecular Surface Area (MolSA), solvent accessible surface area (SASA), and Polar Surface Area (PSA).

Ligand properties were analyzed to evaluate the stability of the selected two compounds, CXB and THY, under the MD simulation. The ligand properties were analyzed based on the RMSD of the ligands, Radius of Gyration (rGyr), Intramolecular Hydrogen Bonds (intraHB), Molecular Surface Area (MolSA), solvent accessible surface area (SASA), and Polar Surface Area (PSA), which were found favorable for all the selected compounds shown in Figure 7.

## 4 Discussion

The enzymes cyclooxygenases (COXs) synthesize autacoids and play key roles in inflammation (Tawfeek et al., 2017). Anti-inflammatory drugs, both steroidal and non-steroidal (NSAIDs), are frequently used in mild to severe inflammatory diseases and (Pilotto et al., 2010), despite their serious adverse effects, as said above. Celecoxib (CXB) is a selective COX-2 inhibitor NSAID that is used to treat different types of arthritis (e.g., osteoarthritis, rheumatoid arthritis, and juvenile rheumatoid arthritis), acute pain, ankylosing spondylitis, painful menstruation, and colorectal adenomas (Abdellatif et al., 2022). On the other hand, ketoprofen (KPN) provides anti-inflammatory, antipyretic, and analgesic effects by reversibly inhibiting COX-1 and COX-2 enzymes (Ahmed et al., 2016; Kang et al., 2020). In this study, THY at 15 and 30 mg/kg exerted KPN-like effects on the experimental animals. It reduced paw edema in animals better than the CXB-42. Further, when combined with these NSAIDs, it exerted better synergistic effects with KPN than CXB. One study reports that THY is more active against the COX-1 enzyme, where the half-maximal inhibitory effect (IC_50_) was determined to be 0.2 µM (Marsik et al., 2005). In contrast, a number of synthesized THY 1,5-disubstituted pyrazole hybrids exerted more potent anti-inflammatory effects than the standards used in a study, where THY (IC_50_ = 0.043–0.068 µM) strongly inhibited the COX-2 enzyme than the CXB and quercetin (El-Miligy et al., 2023). Thus, our study is in agreement with this previously published report.

The housekeeping isoform (constitutive) cyclooxygenase COX-1 plays many important functions in humans, including the production of prostaglandin E_2_ (PGE_2_), which is evidently protective of the gastric mucosa (Williams et al., 1999). On the other hand, the inducible isoform cyclooxygenase (COX-2) is activated by many intracellular and extracellular physiological stimuli, such as lipopolysaccharide (LPS), interleukin (IL)-1, tumor necrosis factor (TNF), epidermal growth factor (EGF), platelet-activating factor (PAF), serum, endothelin, and arachidonic acid (Font-Nieves et al., 2012). Overexpression of COX-2 results in an accumulation of PGE_2_ and is associated with many pathological states, including inflammation. COX-2-induced prostaglandins are also linked with immunosuppressive effects in animals. Studies report that the overproduction of PGE_2_ suppresses both macrophage and natural killer (NK) cell-mediated cytotoxic effects in cancer cells (Williams et al., 1999). A recent meta-analysis performed on the THY’s anti-inflammatory and wound-healing effects suggests that this natural monoterpene phenol acts by reducing IL-1, IL-17, TNF-α, aspartate aminotransferase (AST), myeloperoxidase (MPO), and C-reactive protein (CRP) in experimental animals (Gabbai-Armelin et al., 2022). Therefore, THY, in our present study, may act by inhibiting both COX-1 and COX-2-dependent inflammatory pathways in animals.

Formalin exerts a biphasic response in animals; in Phase I, it directly results in neurogenic pain, while in Phase II, it induces inflammatory reactions through the synthesis of prostaglandins, serotonin, histamine, bradykinin, and cytokines, including IL-1β, IL-6, TNF-α, eicosanoids, and nitric oxide (NO) (Fu et al., 2001). THY at all test doses, standards, and combination groups significantly reduced the paw-licking behavior in mice both in Phase I and II, suggesting that these agents might manage pain at both phases. It has been demonstrated that THY exerts anti-nociceptive and anesthetic (local) effects, possibly via blockade of voltage-operated sodium channels (Haeseler et al., 2002). Thus, the pain management capacity of THY might be due to its inhibitory effects on neurogenic pain mechanisms as well as inflammatory cascades.

Recruitment of leukocytes out of blood vessels or tissues plays an essential role in the development of inflammatory cascades, leading to many inflammatory diseases, including allergies and asthma (Kelly et al., 2007). Egg albumin recruits leukocyte cells at the injection site of paw tissues in animals. It has been observed that endogenous histamine and serotonin have important roles in inflammation processes (Lundberg and Gerdin, 1984). Egg albumin may produce edema by releasing histamine and serotonin (Akindele and Adeyemi, 2007), thus increasing vascular permeability and resulting in the formation of edema in animals (Patil et al., 2019). However, the edema level in this case is significantly lower than the edema observed in other methods, such as carrageenan-induced and formalin-induced models (Barung et al., 2021). In a study, THY inhibited inflammatory edema along with leukocyte migration in carrageenan-induced pleurisy, ear edema, and chemotaxis (*in vitro*) (Fachini-Queiroz et al., 2012). In contrast, Swaggerty et al. (2020) demonstrated that the THY diet significantly increased the functional capability of peripheral blood leukocytes in broiler chicks (Swaggerty et al., 2020). Moreover, THY improved irritable bowel syndrome, where it was found to antagonize endogenous serotonin in a rat model (Subramaniyam et al., 2020). Studies suggest that histamine >50 mg/kg in foods is harmful to humans (Byun and Mah, 2012). One study reports that 1% THY treatment did not stimulate histamine synthesis in broiler meat samples in comparison to the control groups (Zakarienė et al., 2016). Thus, the inhibitory effects on egg albumin-induced edema in chick models observed in our study are in agreement with previous reports on THY.

To date, many important essential oil components, especially monotypes, have been reported for their promising anti-inflammatory effects, such as 1,8-cineole (Juergens, 2014), eucalyptol (Yin et al., 2020), monotropein (Jiang et al., 2020), myrtenal (Rathinam et al., 2022), 6′-*O*-galloylpaeoniflorin (Xu et al., 2022), geraniol (Zou et al., 2022), and so on. Like THY, all these compounds have been shown to possess antioxidant and anti-inflammatory effects on various test systems.

Finding new drugs and developing them takes a long time and a lot of effort. In silico, the docking process has recently gained recognition as one aspect of computerized drug development (Bharatam, 2021). The main advantage of *in silico* drug design is that it significantly reduces the cost of drug development and research. This technique can commit significant resources to every stage of drug manufacture, from concept to completion (Paul et al., 2021). A variety of *in silico* methodologies mix and draw ideas from diverse areas of basic investigation and implementation (Brogi et al., 2020). In this study, our *in silico* results show that THY has a high potential for docking with COX-2 (−6.7 kcal/mol) and COX-1 (−5.9 kcal/mol). THY exhibited non-bonding residues that were similar to KPN with COX-1 including CYS41, LEU152, CYS47, and TYR39 AA residues and the binding location of THY was also similar to that of CXB because both of them bind with the same AA residues including VAL509, VAL335, ALA513, VAL509, LEU338, and PHE504 which formed HB and different HP bonds. Taken together, THY was found to have no selective anti-inflammatory impact in animal models in this study by interacting with same location of COX enzymes where KPN and CXB bind to their respective subtypes of COX.

**FIGURE 8:**
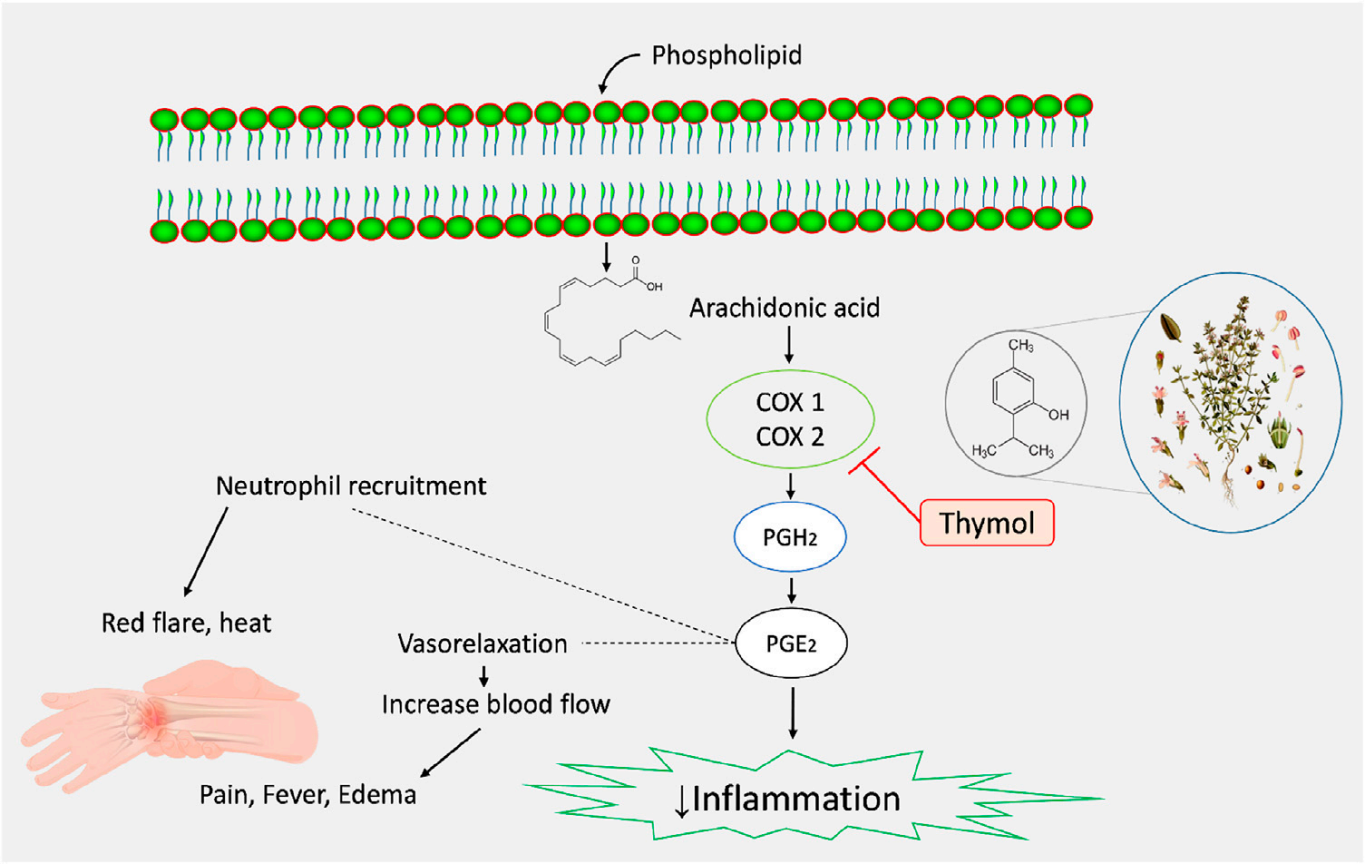
Possible anti-inflammatory mechanisms of action of thymol.

MD simulation analysis is a method of analyzing dynamic trajectories that provides valuable data for evaluating the stability and interactions between proteins and ligands in real-time. Progress is being achieved in the creation of innovative medications by analyzing protein activity and molecular interactions. This technique discerns and detects the alteration in the protein-ligand complex’s structure when subjected to an artificial setting (Sargsyan et al., 2017). The RMSD values between the actual and predicted structures are commonly employed to verify the accuracy of a docked pose predicted by the docking simulation (Fatriansyah et al., 2022). An RMSD analysis can provide insight into whether or not the simulation has reached equilibrium when its final fluctuations cluster around a thermal average structure. Alterations in the sequence one to three Ǻ are deemed to be inconsequential for small, globular proteins (Castro-Alvarez et al., 2017). The protein RMSD values of the selected ligand-protein complexes are below 3 Å, indicating the protein’s acceptable conformation changes. The data from MD simulations showed that the RMSF data confirm that the stability of the compounds with the targeted receptor is determined by the complex’s minimal fluctuation (Fatriansyah et al., 2022). The combined forces of hydrogen bonding and hydrophobic interactions govern the binding stability of complexes. Molecular MD simulations justify the various binding sites and domains of the small molecules to their interested macromolecules (Vaidyanathan et al., 2023). In our study, CXB and THY bind in the same location as COX-2 via forming HB by interacting with AA residues of ARG106, LEU338, and TYR341, which also support the molecular docking study. Figure 8 displays possible anti-inflammatory pathways for THY according to our *in vivo* and *in silico* studies.

## 5 Conclusion

THY reduced formalin-induced paw-licking behaviors dose-dependently in both neuronal and prostaglandin phases in mice. It also reduced paw-licking behavior dose-dependently in egg albumin-induced chicks. THY significantly reduced the paw edema profile in mice and chicks in dose- and time-dependent manners in comparison to the control groups. THY-15 when combined with the standard anti-inflammatory drugs CXB-42 or KPN-42, significantly reduced the paw-licking and edema profiles better than their individual groups, suggesting possible synergistic anti-inflammatory effects with these drugs. However, THY-15 showed better anti-inflammatory effects than KPN-42. Further, *in silico* studies suggest that THY has an elevated binding capacity with COX-2 than COX-1 and the ligand binds in the similar locations of KPN and CXB. The results of MD simulations revealed that THY is stable when it binds with COX-2 and has a better ligand properties. We suppose THY exerts anti-inflammatory effects, possibly through the COX-2 interaction pathway.

## Data Availability

The data will be available upon request from the corresponding authors.
